# Human cytomegalovirus in cancer: the mechanism of HCMV-induced carcinogenesis and its therapeutic potential

**DOI:** 10.3389/fcimb.2023.1202138

**Published:** 2023-06-23

**Authors:** Chuan Yu, Suna He, Wenwen Zhu, Penghui Ru, Xuemei Ge, Kavitha Govindasamy

**Affiliations:** ^1^ Animal Diseases and Public Health Engineering Research Center of Henan Province, Luoyang Polytechnic, Luoyang, Henan, China; ^2^ Department of Pharmaceutical Sciences, School of Basic Medical Sciences, Henan University of Science and Technology, Luoyang, Henan, China; ^3^ School of Light Industry and Food Engineering, Nanjing Forestry University, Nanjing, Jiangsu, China; ^4^ School of Arts and Science, Rutgers, the State University of New Jersey, Newark, NJ, United States

**Keywords:** human cytomegalovirus, mechanism, therapeutic potential, herpesvirus, cancer, virus

## Abstract

Cancer is one of the leading causes of death worldwide. Human cytomegalovirus (HCMV), a well-studied herpesvirus, has been implicated in malignancies derived from breast, colorectal muscle, brain, and other cancers. Intricate host-virus interactions are responsible for the cascade of events that have the potential to result in the transformed phenotype of normal cells. The HCMV genome contains oncogenes that may initiate these types of cancers, and although the primary HCMV infection is usually asymptomatic, the virus remains in the body in a latent or persistent form. Viral reactivation causes severe health issues in immune-compromised individuals, including cancer patients, organ transplants, and AIDS patients. This review focuses on the immunologic mechanisms and molecular mechanisms of HCMV-induced carcinogenesis, methods of HCMV treatment, and other studies. Studies show that HCMV DNA and virus-specific antibodies are present in many types of cancers, implicating HCMV as an important player in cancer progression. Importantly, many clinical trials have been initiated to exploit HCMV as a therapeutic target for the treatment of cancer, particularly in immunotherapy strategies in the treatment of breast cancer and glioblastoma patients. Taken together, these findings support a link between HCMV infections and cellular growth that develops into cancer. More importantly, HCMV is the leading cause of birth defects in newborns, and infection with HCMV is responsible for abortions in pregnant women.

## 1 Introduction

HCMV is an opportunistic pathogen, and its infection is associated with severe morbidities and mortalities in immune-compromised populations, especially organ transplants, AIDS, and cancer patients. HCMV is a pathogen infecting 70-90% of the world population that remains latent over the lifetime of the host after primary infection and reactivates to cause severe disease in immunocompromised patients. HCMV infection in humans causes the progression and development of several cancers, including breast cancer in women (Diaz et al., 2006; Herbein and Kumar, 2014), brain (McFaline-Figueroa and Wen, 2017; Peredo-Harvey et al., 2021) and colorectal cancer (Bai et al., 2016). In addition to this devastating effect, congenital HCMV infection is the leading cause of birth defects. In 1973, the oncogenic potential of CMV was observed when the virus was able to transform hamster embryo fibroblast (HFF) cells and these findings suggested the addition of CMV to the group of viruses capable of inducing malignant transformation (Albrecht and Rapp, 1973).

The oncogenic potential of HCMV is associated with the presence of a number of oncogenes in its genome, such as IE1, IE2 (Shen et al., 1997), US28 (Maussang et al., 2009), and UL76 (Siew et al., 2009). It is believed that there are many other important genes that increase the infectious potency of HCMV in tumor cells, resulting in an aggressive form of malignancy.

**Figure 1:**
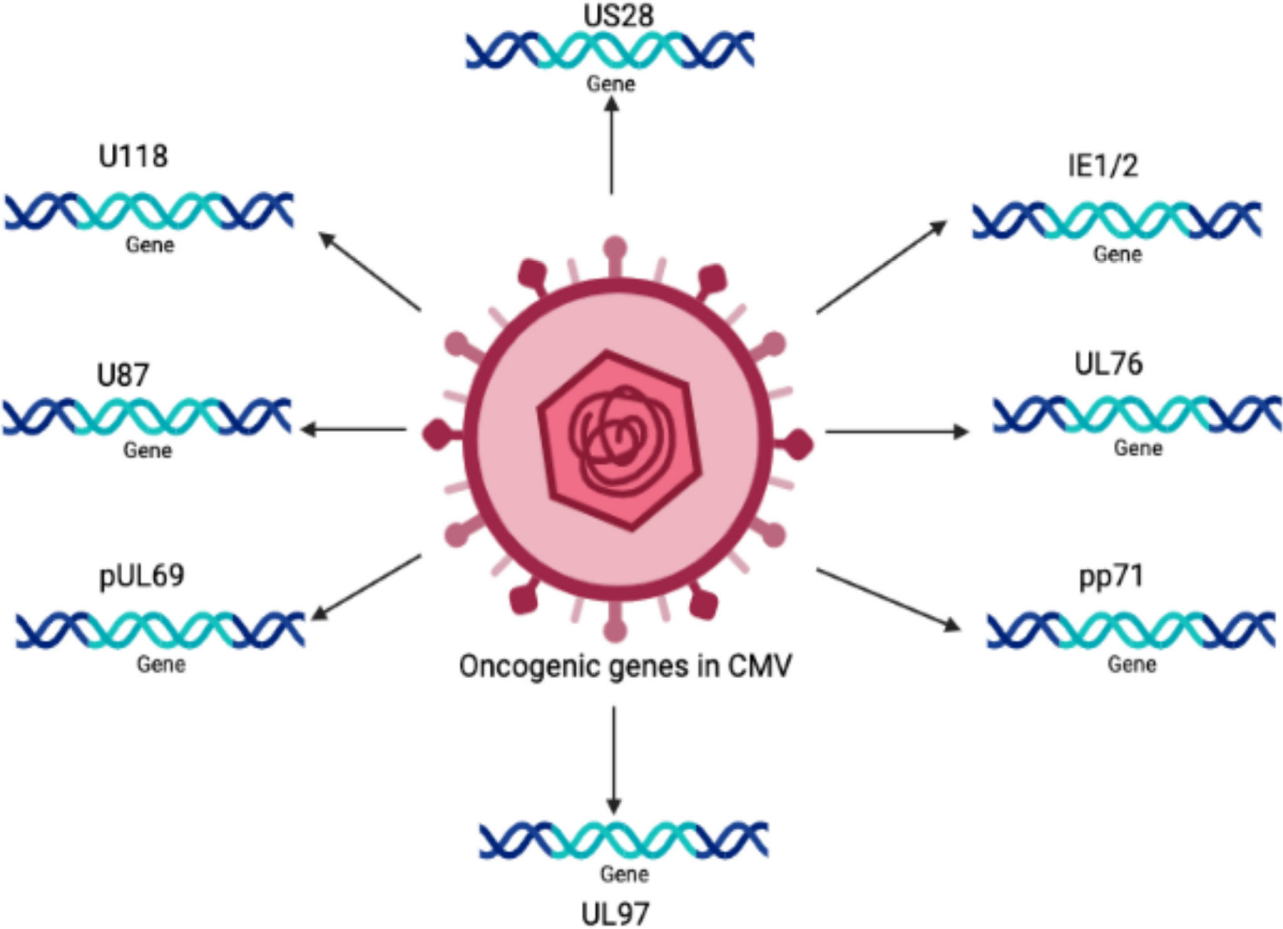
The schematic representation of various oncogenic genes present in the HCMV genome.

Persistent infection with HCMV causes severe complications in immunocompromised individuals and patients with organ transplants. Consequently, HCMV has emerged as a potential candidate for therapeutic purposes and drugs designed to combat these devastating diseases. The relationship between HCMV and cancer is certainly a controversial topic that has been discussed for several decades. Many studies have shown that HCMV is associated with various forms of cancer (Cinatl et al., 1996a; Cinatl et al., 1996b; Michaelis et al., 2009; Moore and Chang, 2010; Michaelis et al., 2011). Moreover, studies have revealed that the HCMV genome encodes numerous oncogenes, including US28, IE1, IE2, and UL76 (Samanta et al., 2003; Joshi et al., 2009), suggesting an important role of HCMV in cancer progression (as shown in **Figure 1**). It has been shown that HCMV infection activates major signaling pathways associated with cancers (Cinatl et al., 2004a). Studies in the past could not confirm that HCMV could transform normal human cells; therefore, HCMV had not been considered a tumor-inducing virus. However, HCMV has recently been reported to induce the transformation and tumorigenicity of cells such as glioblastoma cells *in vitro* experiments (Xing et al., 2016; El Baba et al., 2023). Several elegant studies have shown that HCMV DNA is present in cancer tissues from patients diagnosed with glioblastoma (Mitchell et al., 2008), colorectal cancer (Harkins et al., 2002) and prostate cancer (Samanta et al., 2003). The HCMV genome is ~240 kb and is reported to have approximately 165-252 open reading frames (ORFs). A recent study has shown that the HCMV genome encodes more than 751 transcripts, where it was previously thought to encode only 180 proteins (Stern-Ginossar et al., 2012). This study suggests that there could be many other small RNAs encoded by CMV that may translate into proteins. The authors of this study used ribosome profiling and transcript analysis to redefine the HCMV coding capacity. The author suggested that the HCMV genome uses alternative transcript start sites and allows multiple distinct transcripts to be transcribed from a single genomic locus, resulting in the identification of a large number of previously unidentified proteins. HCMV proteins have been found to interfere with several cellular processes in infected host cells (Cinatl et al., 2004a; Cinatl et al., 2004b; Soderberg-Naucler, 2006). This evidence suggests an important role for HCMV in the development and progression of various forms of cancers.

Several hypotheses have been proposed to explain the role of HCMV in cancer. One mechanism is that HCMV causes cancer by a ‘hit and run’ mechanism (Galloway and McDougall, 1983; Cinatl et al., 1996a). According to this hypothesis, HCMV is required to initiate but not to maintain, the process of transformation. Another study has defined the relationship between HCMV and cancer in terms of “oncomodulation”, where HCMV infects tumor cells and enhances their malignancy, a concept based on experimental findings (Michaelis et al., 2009). It has been postulated that non-coding RNA and viral regulatory proteins that affect processes such as cell survival, proliferation, invasion, and immunogenicity all contribute to HCMV-induced oncomodulation (Michaelis et al., 2009). Therefore, HCMV infection of normal cells can lead to a progressive shift toward a transformed phenotype. Various biological functions such as tumor suppressor proteins, transcription factors, and signaling pathways, are disturbed in transformed cells but not normal cells, which provides an appropriate environment for the oncogenic potential of HCMV (Cinatl et al., 1996a). Persistent productive infection with HCMV is required to catalyze the oncogenic process (Cinatl et al., 1996b; Cinatl et al., 1998). Previously, it has been shown that HCMV infection causes cell cycle arrest and prevents DNA replication in infected cells without affecting viral DNA replication (Castillo and Kowalik, 2004; Sanchez and Spector, 2008). Moreover, it has been shown that a mutated virus is produced after the persistent infection of tumor cells by HCMV. This mutated virus grows slower and produces less progeny than the WT virus (Furukawa, 1984). The virus also contains deletions in its genome, and the IE proteins are of different sizes (Hermiston et al., 1987; Wilkinson et al., 2015). However, the emergence of drug-resistant strains of HCMV urgently requires new targets for therapeutic drug development.

The HCMV-encoded genes described in **Figure 1** and **Table 1** have cellular transformation abilities that result in different types of malignancies, such as breast, brain, colorectal and other forms of cancer. HCMV-encoded IE1 is the first gene to be transcribed and is abundantly expressed during early infection of host cells. The IE-1 protein plays an important role in the establishment of acute infection as well as viral reactivation from latency. Studies have demonstrated that the IE1 gene is expressed in 90% of glioblastoma tumors (Cobbs et al., 2002) and mediates oncogenic effects via interactions with p53 and other tumor suppressor proteins (Lee et al., 2005). HCMV IE1 protein also alters cell cycle progression and is thus responsible for the transformed phenotype of infected cells (Castillo et al., 2000). Additionally, the IE1 gene product inhibits the association of STAT1, STAT2, and IRF9 with IRF-responsive gene promoters (Paulus et al., 2006). Similarly, US28, another important protein encoded by CMV, is associated with G-protein-coupled receptor and shows high homology to several chemokines. The US28 protein also displays oncogenic properties. Studies have shown that US28 promotes tumor formation via its effect on G-protein-coupled receptor, NF-kB, and VEGF signaling pathways (Casarosa et al., 2001; Maussang et al., 2006). Immediate early -2 (IE-2), another important protein encoded in the HCMV genome, also displays the properties of an oncogene and causes cancer progression via its effects on p53 and cell cycle progression (Herbein, 2018). Similarly, UL97 is an essential gene that is responsible for the viral lytic cycle, reactivation of latent virus, late-stage egress of the virus and perturbation of cell cycle progression (Siew et al., 2009). The deletion of the UL97 ORF leads to a complete loss of viral production (Wang et al., 2000). The oncogenic role of UL97 is mediated through its effects on chromosomal aberrations (Siew et al., 2009). A few of the oncogenic genes in the HCMV genome are presented in **Table 1** and **Figure 1**. Several other genes described in **Table 1** and **Figure 1** are also found to have oncogenic properties and cause cancer progression through their effects on the cell cycle and apoptosis pathways.

**Table 1:** A list of HCMV encoded genes that have oncogenic properties and effect several pathways in host cells for cancer progression.

CMV encoded protein	Affected pathways in cells	Oncogenic properties	References
**Immediate early protein-1 (IE-1)**	p53, Rb family proteins, cells cycle alteration, Inhibition of apoptosis, chromosomal aberration	Cellular proliferation, inflammation, genome instability and mutation	(Castillo et al., 2000; Cobbs et al., 2002; Lee et al., 2005)
**Immediate early protein-2 (IE-2)**	p53, cells cycle alteration, PI3K/Akt pathway, TGF-beta pathway	Cellular proliferation, enhanced cell survival,	(Herbein, 2018)
**US28**	GPCR signaling and NF-kB pathways, VEGF pathway, JAK/STAT3 pathway	Tumor growth, angiogenesis, cell survival, cellular proliferation	(Casarosa et al., 2001; Maussang et al., 2006; Herbein, 2018)
**UL69**	Cell cycle	Cellular proliferation	(Hayashi et al., 2000)
**UL97**	Apoptosis	Evading growth suppressor	(Fliss and Brune, 2012)
**pp71**	Apoptosis	Cellular proliferation, genomic mutation	(Taylor and Bresnahan, 2006)
**UL76**	Chromosomal aberration	Genomic instability	(Siew et al., 2009)
**UL 36**	Inhibits caspase-8 Apoptosis	Increased cell survival	(Cinatl et al., 1998; Cinatl et al., 2004b)
**UL 37**	Mitochondria mediated apoptosis	Increased cell survival	(Cinatl et al., 1998; Cinatl et al., 2004b)

## 2 Immunologic mechanisms of HCMV infection in cancer therapy

**Figure 2:**
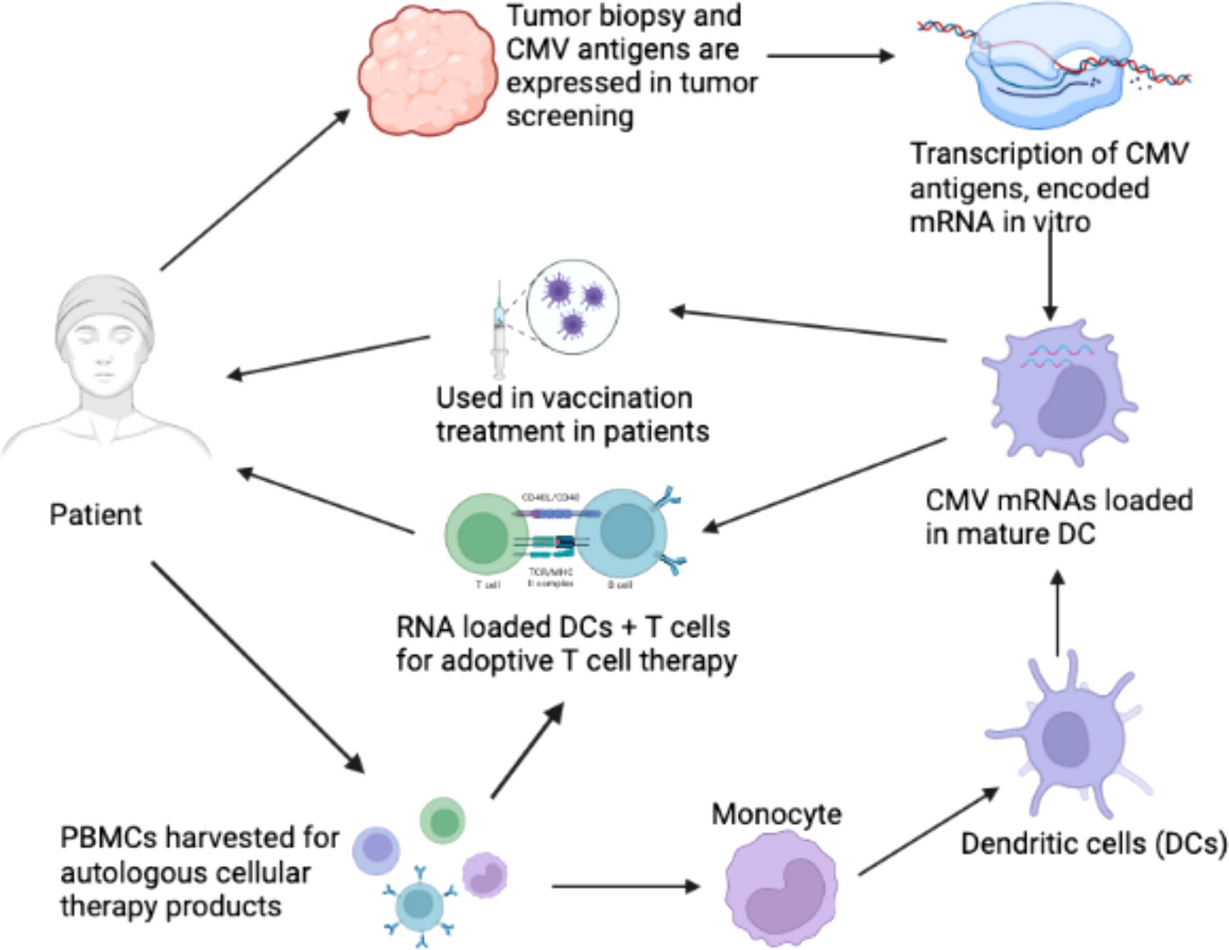
Clinical immunotherapy of glioblastoma multiforme (GBM) via dendritic cells (DCs) loaded with mRNA encoding CMV antigens. 1) Biopsy for screening of cytomegalovirus (CMV) antigen expression (pp65, and IE1) in tumor tissue. 2) Transcription of mRNA encoding CMV antigens *in vitro*. 3) Peripheral blood mononuclear cells (PBMCs) were obtained from leukapheresis to produce T cells for adoptive therapy. 4) Monocytes were isolated from PBMCs. 5) Monocytes differentiated into immature DCs to acquire mature DCs for therapy. 6) Electroporation of CMV antigen-encoding mRNA to mature DCs. 7) DCs loaded with CMV antigen-encoding RNA in vaccination strategies for the treatment of GBM. 8) DCs loaded with CMV antigen-encoding RNA were used to stimulate autologous T cells for adoptive T cell therapy in GBM (Nair et al., 2014b).

It has been suggested that CMV functions as an important immunotherapeutic target in cancer therapy for cancers such as glioblastoma (Schuessler et al., 2014b; Batich et al., 2017). Some studies have provided evidence that CMV antigen-pulsed autologous dendritic cells, autologous CMV-specific T cells, CMV-targeting CARs, and peptide vaccines could be promising approaches for glioblastoma treatment (Schuessler et al., 2014b; Lawler, 2015). It has been reported that HCMV could be differentially detected in glioblastoma rather than normal brain tissues, making CMV antigens a potential target for glioblastoma multiforme (GBM) treatment. The expression of CMV antigens may provide a feasible solution for immune treatment of HCMV-related cancers. HCMV has developed several strategies to escape from the immune surveillance of the host. For the treatment of HCMV infection-related cancers, HCMV-directed immunotherapy has been used as a novel approach, and some related clinical trials have been conducted (Schuessler et al., 2014a). For example, pp65 has been used as an immunological target for the treatment of HCMV infection. As shown in **Figure 2**, generated immature DCs were transfected with the CMV gene encoding mRNA and then matured using a standard maturation cocktail of GM-CSF and IL-4 with TNF-α, IL-1β, IL-6, and PGE2 (Nair et al., 2014b). It has been indicated that patient-derived CMV pp65-specific T cells recognize autologous target cells and can be used as an effective way to treat GBM (Nair et al., 2014a). **Figure 2** explains that CMV specific autologous T cells and autologous DCs can be used in immunotherapy for GBM patients. The autologous DCs were isolated and electroporated with CMV pp65 mRNA. The pp65 mRNA was synthesized *in vitro* and then electroporated into the mature DCs. The DCs that are used in vaccination only contain pp65 mRNA, while the DCs used in adoptive T cell therapy contain pp65 mRNA and are administered with pp65 specific T-cells. The T cells and DCs are isolated from the single patient who is undergoing for GBM immunotherapy.

Host immune evasion by HCMV suggests its implication in the development of a tumor microenvironment. The genes encoded by HCMV can modulate an innate immune response, such as the interferon response (Amsler et al., 2013). Moreover, HCMV genes play roles in modulating the intrinsic and extrinsic pathways of apoptosis (Fliss and Brune, 2012). One of the mechanisms used by HCMV to evade the host immune response is apoptosis, which is described in more detail in section 3.2. HCMV immediate early gene product (IE1) plays an important role in immune system evasion via its effects on the STAT1 and STAT2 binding with the promoter of IFN-responsive genes (Paulus et al., 2006). Similarly, the IE2 gene interferes the association of NF-kB with the IFN-β promoter (Taylor and Bresnahan, 2006). More importantly, HCMV encodes several viral homologs of cytokines such as UL146 (IL-8 like) and UL111a (vIL10) (Jackson et al., 2017). HCMV vIL10 is secreted by tumor cells and is associated with the presence of TAMs in associated cancers. Furthermore, HCMV evades the immune system by degrading the major histocompatibility complex class-1 (MHC-1) heavy chain through the endoplasmic-reticulum-associated protein degradation (ERAD) pathway, which are mediated by viral US-1 and US11 encoded proteins (Tortorella et al., 2000). The US2 glycoprotein can inhibit the translocation of MHC-II molecules, thus allowing the virus to evade the CD4+ T cell response.

## 3 HCMV infection modulates various pathways in cancer cells

### 3.1 Cell cycle

**Figure 3:**
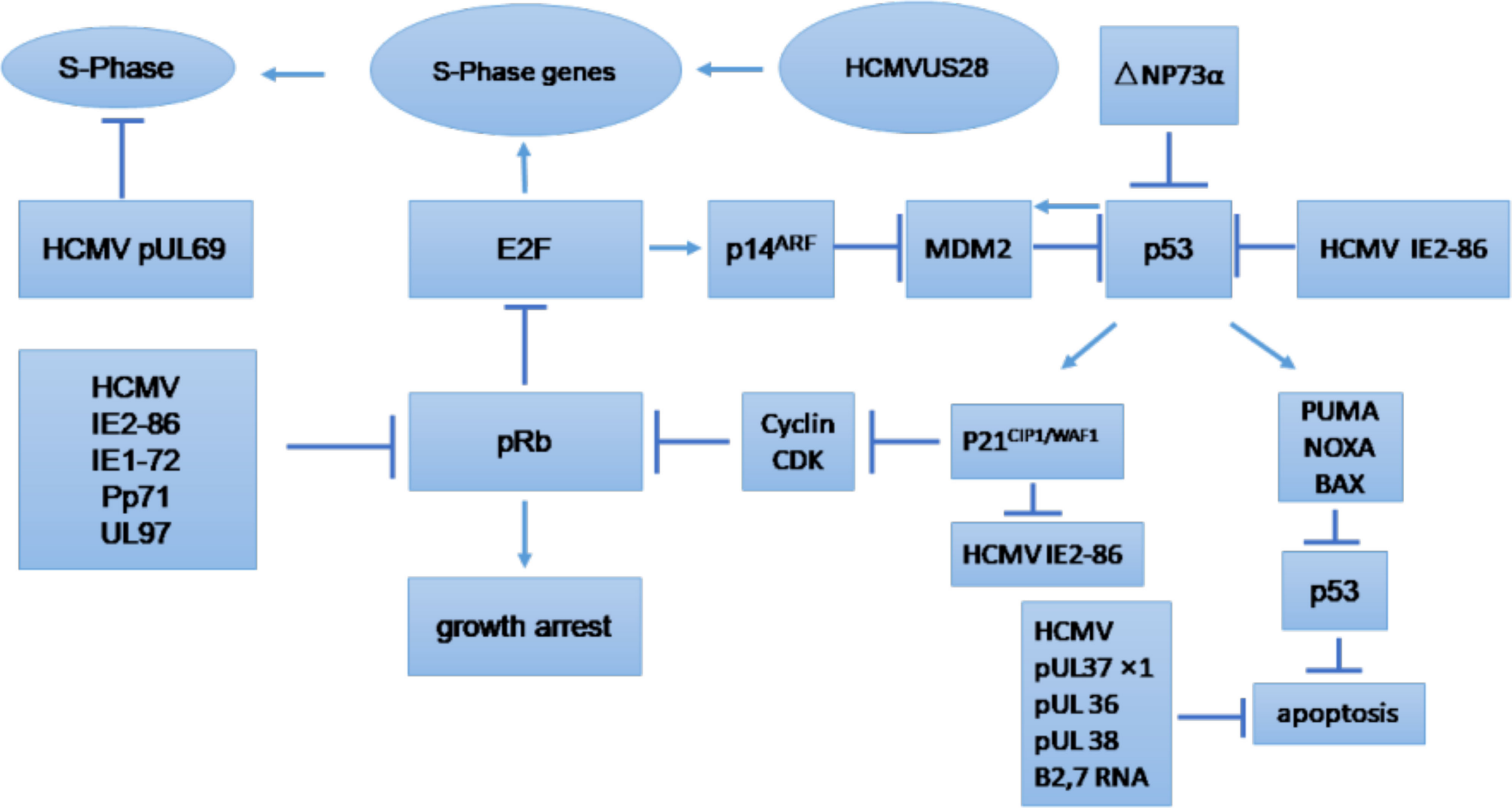
Tumor cell cycle regulation and/or apoptosis caused by HCMV. CDK, cyclin-dependent kinase; E2F, E2F transcription factor; MDM2, mouse double minute 2. (Michaelis et al., 2009).

The cell cycle is an essential process that involves series of events that take place in all mitotic cells. It has been suggested that HCMV-encoded proteins involved in the replication of viral DNA can block the host cell cycle process (Castillo and Kowalik, 2004; Sanchez and Spector, 2008). The major effects of HCMV on regulation of the tumor cell cycle and/or apoptosis are shown in **Figure 3**, which provides general information on the possible molecular mechanism of HCMV infection in tumor cells (Michaelis et al., 2009). The influence of HCMV on the cell cycle of cancer cells is commonly deregulated, and this process may depend on the internal conditions of the tumor cells. Some studies have indicated that HCMV infection could have an antiapoptotic effect through the IE proteins (Cinatl et al., 2004a). The HCMV immediate early-1 protein with a molecular weight of 72 kDa (IE1-72), and, the immediate early-2 protein with a molecular weight of 86 kDa (IE2-86), could reduce Rb family protein (pRb, p107, and p130) activities in infected host cells and thus prevent cellular entry into S phase. The cell cycle in cancer is typically dysregulated, and the effect of proteins expressed by HCMV genomes is mostly dependent on the behavior of the tumor cells (Cinatl et al., 2004a). Moreover, it was found that the HCMV IE1-72 protein could significantly affect important oncogenic signaling pathways in glioblastoma cells, which indicates an important role of HCMV in the induction of glioblastoma (Cobbs et al., 2002; Cinatl et al., 2004a). HCMV infection in U87 and U118 glioblastoma cells reduced the expression of Rb and p53 family proteins (including p53, p63 and p73), which could be a key factor for IE1-72-induced cellular proliferation. An elegant study confirmed that IE1-72 expression in HCMV-infected LN229 and U251 glioblastoma cells was altered and that the p53 family proteins in infected cells were increased, leading to cell cycle arrest (Cobbs et al., 2008). Recently a significant increase in cell proliferation in HT29 and SW480 cells after 24, 48 and 72 h of HCMV infection was reported, indicating that HCMV infection could affect the cell cycle of CRC-derived HT29 and SW480 “stem-like” cells (Teo et al., 2017). HCMV US28 chemokine receptor increases the expression of cyclin D1 protein and mediates the transition from the G1 to the S phase of the cell cycle (Maussang et al., 2009; Soroceanu et al., 2011). The G-protein coupling activity of US28 is essential for enhanced cell cycle progress and promotes the transformation of infected cells (Maussang et al., 2009; Soroceanu et al., 2011). Additionally, US28 promotes an angiogenic phenotype via its effect on VEGF, which enhances the oncogenic phenotype (Maussang et al., 2009; Soroceanu et al., 2011).

### 3.2 Apoptosis

Programmed cell death (apoptosis) occurs in all multicellular organisms and is a process that is often evaded in cancer. Apoptosis is an antiviral response that is detrimental to HCMV growth cycle because it kills infected cells and thus prevents persistent HCMV infection. Therefore, HCMV have several strategies to prevent premature or programmed cell death of infected host cells to survive and spread to adjacent cells. HCMV has been reported to prevent apoptosis of cancerous cells, and this anti-apoptotic effect is mediated by IE proteins via both p53-dependent and p53-independent pathways (Tsai et al., 1996; Tanaka et al., 1999; Nair et al., 2014b). Moreover, HCMV-encoded UL36-UL38 also exhibit anti-apoptotic functions. UL36 is an inhibitor of caspase activation and inhibits Fas-mediated apoptosis (Skaletskaya et al., 2001). UL37 is another HCMV-encoded protein that also serve an anti-apoptotic role by preventing the recruitment of proapoptotic factors, Bax and Bak, to mitochondria (Goldmacher et al., 1999; Norris and Youle, 2008). Another gene in HCMV is pUL38, which encodes a cell death inhibitory protein and enhances viral replication by preventing premature cell death (Terhune et al., 2007). ATF5 is an anti-apoptotic factor in glioma cells. HCMV infection of U87 cells has been found to increase the cellular proliferation of infected cells by upregulating the expression of ATF5 and increasing the Bcl-2 to BAX ratio (Wang et al., 2014). **Table 2** describes the function of HCMV-encoded proteins that play important roles in preventing the apoptosis of infected host cells. These proteins form the basis of HCMV-mediated tumor progression by altering the tumor environment to promote cancer cell growth.

**Table 2:** The role of HCMV encoded proteins in preventing apoptosis of infected host cells.

HCMV encoded genes	Role in apoptosis	References
**IE1 and IE2**	Prevent apoptosis through Akt mediated pathways	(Yu and Alwine, 2002; Cobbs et al., 2008)
**UL38**	Encodes cell death inhibitory protein and prevent apoptosis	(Terhune et al., 2007)
**UL36**	Prevent apoptosis through pro-caspase 8	(Skaletskaya et al., 2001)
**UL37**	Prevent apoptosis by sequestering proapoptotic protein bax	(Goldmacher et al., 1999; Norris and Youle, 2008)
**US2, US11**	Bind to MHC-1	(Rehm et al., 2002)
**US3**	Prevent transport of MHC-1 from ER- Golgi	(Noriega et al., 2012)
**US6**	Prevent peptide translocation into ER by TAP	(Ahn et al., 1997)

### 3.3 Role of CMV in apoptosis and necroptosis of noncancerous cells

Apoptosis is a programmed cell death that may be activated by cell stress (intrinsic pathway) or cytokines (extrinsic pathway). Studies have shown that HCMV encodes a UL36 protein that inhibits caspase-8 activation (Skaletskaya et al., 2001) or M36 homolog in MCMV (Menard et al., 2003). Moreover, it has been found that both HCMV (UL36) and MCMV (M36) can protect infected cells from CD8+ T cells by preventing apoptosis (Chaudhry et al., 2020). Necroptosis is a regulated cell death characterized by cell lysis followed by the secretion of damage-associated molecular patterns. Inhibition of apoptosis function usually makes cells more susceptible to necroptosis (Mocarski et al., 2011). Studies have shown that inhibition of caspase-8 promotes extrinsic apoptosis to the necroptotic pathway (Holler et al., 2000). Enhanced necroptosis is an anti-viral response mediated by host cells (Nailwal and Chan, 2019). The biological function and molecular mechanism of necroptosis is still not clear. Studies have shown that necroptosis plays an important role during microbial infection, initiated in response to death receptors in TNFR, TLR4, and TLR3. Necroptosis is a different cellular process than apoptosis (Galluzzi et al., 2017). Studies have shown that receptor-interacting protein kinase 3 (RIPK3) is a main adaptor for necroptosis. RIPK3 is a pro-necrotic serine-threonine kinase and plays a critical role in necroptosis. RIPK3 also has a RIP homotypic interaction motif (RHIM) at its c-terminus and plays a critical role in necroptosis by involving in protein-protein interaction during microbial infection (Sun et al., 2002). Necroptosis has also an important role during viral infection (Cho et al., 2009; Berger and Danthi, 2013). An elegant study has shown that murine CMV express a protein M45 that acts as an inhibitor of RIP activation suggesting a biological role of necroptosis in host defense (Upton et al., 2010; Upton et al., 2012). These studies suggest that CMV specifically inhibits necroptosis to develop productive infection of host cells. Moreover, human cytomegalovirus has been found to inhibit necroptosis by suppressing the RIP3(Omoto et al., 2015). This study has found that HCMV-infected cells are susceptible to TNFR1-dependent necroptosis. More particularly, it was found that HCMV IE1 plays an important role in the suppression of necrosis (Omoto et al., 2015). Moreover, MCMV has been found to suppress apoptosis and necroptosis to establish latency and infection in host cells (Terhune et al., 2007; Chaudhry et al., 2020). A study found that CMV inhibits TNFa-induced necroptosis in cardiomyocytes (Yadav et al., 2023). HCMV merlin strain infection degrades mixed lineage kinase domain-like protein (MLKL) which is a key regulator of cellular necroptosis. Authors reveal that pUL36 was essential to degrade MLKL and suppress necroptosis (Fletcher-Etherington et al., 2020). The presence of CMV DNA in the cytoplasm of infected cells can activate necroptosis (Zhang et al., 2016). All this accumulating evidence suggests that CMV infection can inhibit necroptosis of the infected cell to promote viral replication. In addition to apoptosis, necroptosis induction represents an alternate pathway to remove cancerous cells. Studies have suggested that necroptosis increase anti-tumor immunity by enhancing the immunogenicity of dead tumor cells (Yatim et al., 2015; Yang et al., 2016). Cancerous cells are found to have dysregulated necroptosis pathways and downregulation of RIPK3, a key regulator of necroptosis was observed in different types of human cancer including colorectal cancer (Yan et al., 2018), breast cancer (Koo et al., 2015), and acute myeloid leukemia (Nugues et al., 2014).

### 3.4 Cancer cell invasion, migration, and adhesion

Cell invasion, migration, and adhesion to the endothelium are very important events that take place during the process of tumor formation, and it has been reported that HCMV infection could have some association with these processes. Important cellular signaling events such as phosphatidylinositol-3 kinase/protein kinase (PI3K/AKT) and mitogen-activated protein kinase (MAPK) pathways, are responsible for cell growth and proliferation and are known for their essential role in tumor formation (Mayer and Arteaga, 2016). Studies have shown that the HCMV gB glycoprotein activates the PI3K/AKT pathway in infected cells and is responsible for increased growth, cell survival and migration of cancer cells (Cobbs et al., 2014). HCMV genome encodes many proteins that promote cancer cell activity. The HCMV IE1 protein was reported to induce NF-kB expression and was responsible for the activation of cell survival pathways in tumor cells (Yurochko et al., 1995). More importantly, HCMV-encoded protein UL38 has been reported to block the function of tuberous sclerosis protein 2 (TSC2), dysregulate the mTOR pathway, and induce survival as well as adhesion of infected cells (Moorman et al., 2008). Teo et al. confirmed that HCMV infection could play a key role in colorectal cancer cell proliferation, migration and epithelial-mesenchymal transition (EMT) (Teo et al., 2017). Ding et al. demonstrated the role of integrins and PDGFRs in inducing glioma cell motility and invasiveness via the αvβ3 integrin/PDGFRα pathway (Ding et al., 2003). Meanwhile, cell invasion and migration induced by HCMV infection could also be enhanced via activation of α5β1, COX-2, and extracellular matrix proteases in some tumor types (Tsujii et al., 1997; Tsujii et al., 1998; Scholz et al., 2000). HCMV encoded protein US28 has been reported to play a critical role in migration and invasion of virus-infected cells. The US28-induced invasive and angiogenic phenotype of glioblastoma multiforme (GBM) cells is due to dysregulation of WNT signaling and uncontrolled of cell proliferation (Streblow et al., 1999). Furthermore, US28 promotes cell migration via RANTES and MCP-1 (Streblow et al., 1999). This evidence suggests that HCMV promotes the migration, adhesion, and proliferation of infected cells, which are properties of metastatic tumors. HCMV infection of tumor cells further decreases their adhesiveness with neighboring cells and increases their invasive property.

### 3.5 Macrophages and fibroblasts

Macrophages and other monocytes represent the first line of defense against pathogens and are activated by a T helper-1 (Th-1) type of response via IFN-γ secretion. Macrophages constitute the major reservoir for HCMV, while fibroblasts are the most widely used cell type to experimentally grow HCMV. Macrophage differentiation is inhibited by HCMV in order to replicate and grow optimally in this cell type. An intact ULb’ region has been identified in the genome of HCMV that allows the virus to grow in macrophages, endothelial, and epithelial cells (Nogalski et al., 2013; Murrell et al., 2016; Yue et al., 2016). However, this ULb’ region has been reported to be absent in laboratory strains of HCMV and is responsible for the reduced growth of the virus in macrophages and epithelial cells (Murphy et al., 2003). Clinical strains of HCMV produce a persistent and productive infection in macrophages and endothelial cells, causing the activation and secretion of cytokines and chemokines (Bayer et al., 2013). HCMV modulates several cellular signal transduction events in infected macrophages, particularly via the HCMV-encoded glycoprotein gB that can interact with epidermal growth factor receptor (EGFR) and activate the apoptosis suppressor Akt (Cojohari et al., 2016). Macrophages that are associated with the tumor, called “tumor-associated macrophages” (TAMs), have been detected in HCMV-associated tumor (Mantovani et al., 2017). The TAMs exhibit an M2 phenotype and secrete cytokines such as IL-10 and TGF-beta, which help HCMV immune evasion (Tang, 2013). These cytokines help tumor cells migrate in breast and favour the establishment of microenvironments supportive of the associated tumor (Singh et al., 2014), thereby aiding in the escape from immune surveillance. Furthermore, HCMV infection of macrophages and fibroblasts leads to NF-kB activation through UL55 (gB) (Yurochko and Huang, 1999). In addition to NF-kB, p52/BCL3 is also activated following macrophage infection by HCMV (Khan et al., 2009). It has been reported that CMV-infected fibroblasts have an increased p16 mRNA level that leads to cell cycle arrest (Zannetti et al., 2006). These signaling events modulated by HCMV favor optimal viral growth in macrophages and fibroblasts.

## 4 HCMV infection-related cancers in humans

### 4.1 HCMV and breast cancer

**Figure 4:**
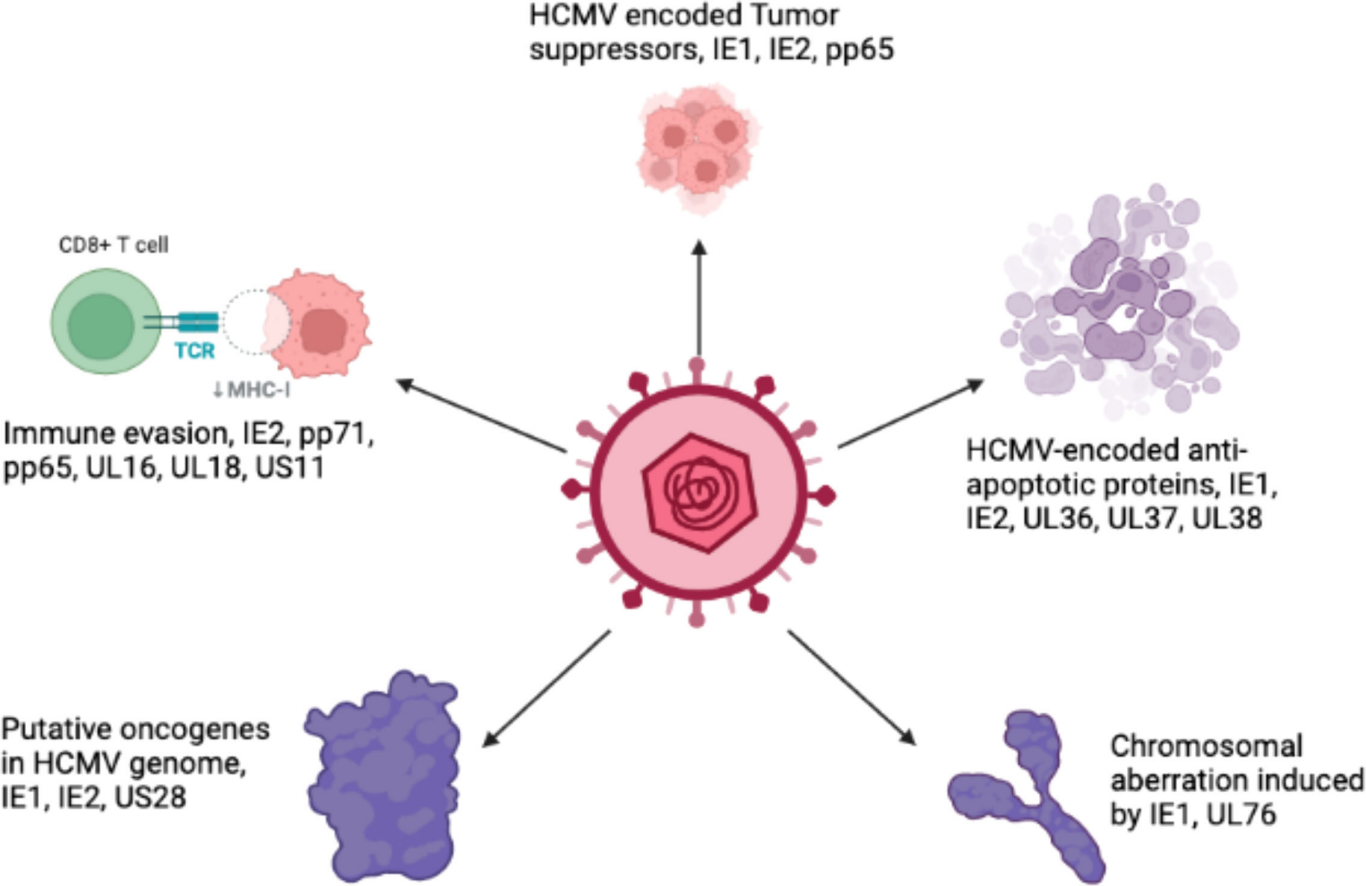
Schematicrepresentation of various pathways in cells modulated by HCMV proteins.

Breast cancer is the leading cause of cancer-related deaths in women and impacts over 1.5 million individuals every year (Joshi et al., 2009; Harkins et al., 2010). The majority of breast cancers originate in the glands that produce milk, and are consisted of several lobules and ducts. Several studies have identified a relationship between breast cancer and many viral infections, including Epstein-Bar virus (EBV), human papilloma virus (HPV), mouse mammary tumor virus (MMTV), human herpesvirus and HCMV (Melana et al., 2010). The oncogenic traits of HCMV rely on the presence of several oncogenes. These genes, along with other essential genes, increase the infectious potency of HCMV in tumor cells, resulting in an aggressive form of malignancy. The HCMV-encoded US28 protein has been found to play a crucial role in G-protein-coupled receptor (GPCR) modulation in HCMV-infected cells (Lee et al., 2017b). The US28 protein can stimulate ligand-specific and nonspecific pathways in host cells that are crucial for HCMV survival (Molleskov-Jensen et al., 2015). HCMV infection modulates various cellular pathways in infected breast cells, as described in **Figure 4**. The HCMV-encoded proteins that prevent apoptosis are IE1, IE2, UL36, UL37, and UL38. These proteins play important roles in HCMV-induced carcinogenesis by favoring virus growth within infected cells. The HCMV-encoded proteins that participate in viral-mediated oncomodulation and their affected pathways are described in **Table 1**.

HCMV has been found in the breast milk of more than 90% of lactating women that are seropositive for anti-HCMV antibodies (Asanuma et al., 1996) and is considered to be the leading cause of birth defects in newborns. Moreover, CMV viral titers detected in 30-40% of children below the age of one year (Asanuma et al., 1996). An elegant study demonstrated a correlation between HCMV infection and breast cancer in which, 399 women with invasive breast cancer and matching healthy volunteers were analyzed. In this study, it was found that the level of IgG antibodies against HCMV was higher in women with breast cancer than that in the control group. In addition, data from Cobb’s laboratory provided more direct evidence of the breast epithelium acting as an important reservoir for HCMV in humans. In another study, using immunohistochemistry, *in situ* hybridization, and PCR, the presence of HCMV antigens was detected in glandular epithelium 17/27 (63%) of normal adult breast tissues (Harkins et al., 2010). In this study, HCMV expression was also detected in the 31/32 (97%) of neoplastic epithelium patients who were diagnosed with ductal carcinoma *in situ* (DCIS) and infiltrating ductal carcinoma (IDC) (Harkins et al., 2010). The exact role of CMV in breast cancer is unknown. A recent study has shown that CMV-encoded IE proteins, late antigens, DNA and RNA were detected in biopsy samples from breast cancer patients (Rahbar et al., 2017). A total of 81 biopsy samples from breast cancer patients were studied in this report and it was observed that IE proteins were present in 77% samples (Rahbar et al., 2017). Moreover, the expression of the estrogen receptors and progesterone receptors was found to be inversely correlated with HCMV infection. Furthermore, HER2 expression was also reported to be reduced in HCMV-infected tissue samples from breast cancer patients (Rahbar et al., 2017). A new strain named HCMV-DB (accession number KT959235) was isolated from a 30-year-old pregnant woman (Moussawi et al., 2018). HCMV-DB can infect primary macrophages and was found to upregulate the Bcl-2 proto-oncogene. The genome of HCMV-DB was found to be closely related to that of other laboratory strains of HCMV, with more similarity to the Toledo strain (Lurain et al., 2006; Cunningham et al., 2010). The HCMV-DB strain infects human mammary epithelial cells (HMECs) and displays an ER-/PR-/HER2-(triple-negative) phenotype and presents oncogenic traits (Moussawi et al., 2018). In this study, it was found that HCMV-DB strain is implicated in breast cancer development. Transcriptomic profiling of HCMV-DB-infected HMECs confirmed the upregulation of numerous oncogenes, activation of prosurvival genes, upregulated markers of cell proliferation, and tumor formation in breast cells (Moussawi et al., 2018).

In a recent study, it was found that 70 of 146 (47.9%) breast cancer samples were positive for HCMV IE proteins and CMV late antigens were detected in 78/146 (53.4%) samples from breast cancer patients (Cui et al., 2018). More importantly, HCMV IE and LA proteins were found to be expressed in 92.6% (62/68) of metastatic sentinel lymph node tissues (Cui et al., 2018). These accumulating evidence indicates that CMV can play a role in breast cancer progression.

### 4.2 HCMV and GBM

GBM is an aggressive human brain cancer that occurs primarily in the central nervous system and is associated with poor prognosis and survival. Additionally, HCMV infection often co-occurs with GBM (Bergenheim et al., 2007; Affronti et al., 2009). Cobbs et al. reported that HCMV nucleic acids and proteins were present in high percentages of low-and high-grade malignant gliomas (Cobbs et al., 2002). The levels of early and late HCMV-encoded protein products were detected in tumor tissues from glioblastoma patients. For example, immunohistochemical assays indicated that the HCMV-encoded immediate early protein IE1-72 protein was present in 27 malignant glioma surgical specimens from nonimmunocompromised patients (Cobbs et al., 2002). HCMV encodes a G-protein-coupled chemokine receptor, US28, which is considered to play an important role in GBM progression. US28 regulates the VEGF promoter activity via the Gαq, Gβγ, p38, and p44/42 kinases and induces VEGF gene upregulation, which is one of the major factors for tumor progression in GBM patients (Maussang et al., 2006; Maussang et al., 2009). Data indicates that transgenic mice over-expressing US28 in intestinal epithelial cells had a hyperplastic intestinal epithelium and experienced tumor progression (Bongers et al., 2010). Interestingly, US28 inhibits glycogen synthase-3β and increases β-catenin accumulation, resulting in the increased expression of Wnt target genes involved in the cell proliferation (Bongers et al., 2010). The US28 protein has been reported to play an essential role in cell migration and invasion of CMV-infected cells. It has been suggested that the US28-induced invasive and angiogenic phenotype of Glioblastoma multiforme (GBM) is caused by the dysregulation of WNT signaling and cell proliferation (Streblow et al., 1999). Moreover, US28 was found to promote cell migration via RANTES and MCP-1 (Streblow et al., 1999). This suggests US28 is an oncogenic protein and is implicated in the progression of GBM. Numerous studies have aimed to illustrate the potential correlations between GBM progression and the presence of HCMV genomes, RNA, and proteins (Solomon et al., 2014). Yang, et al. investigated the presence of several key genes (such as HCMV UL73, and HCMV UL144, which were identified with PCR), RNA, and proteins from HCMV in 116 GBM patients (Yang et al., 2017). The HCMV UL73 gene was detected in a very low proportion of GBMs (7.8%). This could be due to the lack of an optimized method for detecting CMV antigens and antibodies in malignant tissues. There is, however, a known problem in detecting the virus with PCR and sequencing methods, while virus proteins and DNA are readily detected by optimized staining protocols or *in situ* hybridization assays. Using a murine CMV in a syngeneic GBM model, a recent study has reported that CMV markedly increases tumor growth and leads to a significant reduction in overall survival. This study identified that the PDGF-D factor is responsible for the CMV-mediated angiogenesis and tumor growth (Krenzlin et al., 2019). CMV reactivation in the brain after radiotherapy has been reported and presents a major threat to GBM patients. A recent study found that out of 50 patients, 32 developed viremia after 28 days of radiotherapy treatment, and 15 required treatments for CMV-mediated encephalopathy. These results suggest that the identified CMV represented the reactivation of the virus after radiotherapy rather than a primary infection in GBM patients (Goerig et al., 2016). Glioblastoma patients showed overall survival compared to control (24 versus 14 months) when treated with the anti-CMV prodrug of ganciclovir (Soderberg-Naucler et al., 2013; Stragliotto et al., 2015). Signal transducer and activator of transcription 3 (STAT3), which could be activated by HCMV infection, plays important roles in glioma mesenchymal phenotype, angiogenesis, cell survival, and immune- suppression/immune evasion (Ferguson et al., 2016). This could serve as a potential therapeutic target to reduce the GBM growth (Yang et al., 2017).

### 4.3 HCMV and colorectal cancer

Colorectal cancer (CRC) is a life-threatening disease that is the third-leading cause of cancer-related deaths. In the past decade, scientists have made many efforts on finding the mechanisms of this disease and developing effective treatments. CRC pathogenesis is associated with various environmental components, such as lack of exercise, high-fat diet, smoking, and the environment in developed countries. However, scientists have recently been attracted to the contribution of microorganisms such as HCMV to CRC pathogenesis. Many studies have confirmed the presence of HCMV DNA in CRC tumor tissues, indicating an unidentified role of HCMV in the development of the CRC (Huang and Roche, 1978; Mariguela et al., 2008; Lee et al., 2017a). It was reported that HCMV infection could enhance cell proliferation, migration, and upregulation of epithelial-mesenchymal transition (EMT) markers in both CRC and CRC-derived cells (HT29 and SW480 cells). These results showed that WNT signaling (associated with the proliferation and migration of CRC cells) was upregulated by 6-fold in HCMV-infected cells (Teo et al., 2017). While the association with CMV infection was observed in both colorectal cancer and ulcerative colitis (14/21, 66.7%), the results indicated that CMV infection was more frequent in ulcerative colitis (12/21, 57.1%) than that in colorectal cancer tissue (2/14, 14.3%) (Mariguela et al., 2008). **Table 3** describes HCMV-associated cancers and related pathways modulated by CMV.

**Table 3:** Some HCMV-infection related malignancies in humans.

Some of the HCMV associated cancers	Key pathways modulated by HCMV	References
Breast cancer	PI3K-Akt, Ras and Myc pathways, JAK/STAT pathway, Wnt/beta-catenin, MAPK-ERK	(Herbein and Kumar, 2014; Guo et al., 2015)
Brain cancer	VEGF gene up-regulation NF-kB activation, EGFR, ERK pathways	(Casarosa et al., 2001; Cobbs et al., 2007)
Colorectal cancers	WNT signaling (associated with proliferation and migration of CRC cells)	(Teo et al., 2017)
Prostate cancers	protein kinase A (PKA) pathway activation	(Moon et al., 2008)
Ovarian cancer	inflammatory factor 5-lipoxygenase (5LO) associated antiapoptotic signaling pathways activation	(Rahbar et al., 2021)
Sarcoma mucoepidermoid cancer	COX/AREG/EGFR/ERK signaling pathway	(Melnick et al., 2012)

### 4.4 Cytomegalovirus and muscle cancer

Carcinoma of muscle is rare compared to other tissue cancers like bone, lung, pancreas, and liver. Smooth muscle cells are frequently infected by CMV (Persoons et al., 1994; Sinzger et al., 1995; Gredmark-Russ et al., 2009). Majorly, aortic smooth muscles are targeted or infected by CMV. People with immunodeficiency, especially solid organ transplant patients face the problem of neoplasm. Post-organ transplantation, smooth muscle tumors are developed due to viral infection (Anbardar et al., 2022). Biopsy of the smooth muscle tumor and PCR analysis confirmed the presence of EBV and CMV DNA (Anbardar et al., 2022). In another study, about 43% of heterozygous Trp53 mice develop pleomorphic rhabdomyosarcomas after infection with murine CMV compared with uninfected where only 3% of mice develop the tumor (Price et al., 2012). In this study, mice at the age of 2 days were infected with murine CMV and 43% of infected mice develop smooth muscle tumors by the age of 9 months (Price et al., 2012). A case study has shown that an AIDS patient with pulmonary inflammatory myofibroblastic tumor (PIMT) had CMV as confirmed by a serological study on the tumor tissue suggesting a role of CMV in this type of tumor (Cambrea et al., 2014). Serological tests were negative for mycoplasma and Epstein-Barr virus (EBV). PIMT is a rare disease that occurs mainly in younger people. Moreover, a case of polymyositis was also reported in patients with CMV infection. A 17-year-old girl had symptoms of muscle weakness, myalgia, and fever with increased creatine kinase. Histopathological analysis confirmed polymyositis and the patient was seropositive for CMV infection (Maeda et al., 2000). In another case study, a patient with primary CMV infection was diagnosed with altered skeletal muscle structure, myalgia, and increased serum creatine kinase (CK) (Ripolone et al., 2022). Muscle biopsy showed an increased NADH expression. Structural analysis of muscle tissue showed an altered sarcomere structure with thin filaments. Immunohistochemistry analysis of muscle biopsy tissues confirmed the altered structure of actin, αβ-crystallin, and desmin proteins (Ripolone et al., 2022). CMV was also reported to be associated with rhabdomyolysis (Hughes and Hunt, 1984). Studies have confirmed that CMV can reduce the expression of endothelin-1 in smooth muscle which may lead to deformed muscular structure formation (Yaiw et al., 2021). Interestingly, CMV infection also has been found to be associated with dermatomyositis (DM) patients. Dm is an autoimmune disease characterized by muscle weakness (Yaiw et al., 2021). There is not enough evidence that proves that CMV can cause DM. CMV is a major opportunistic pathogen in immunosuppressive people. A case study found that biopsy muscle samples of neuromyositis patients were found to be positive for CMV staining (Oumerzouk et al., 2013). In addition to cancer, studies have shown that viral infections are associated with mild myopathies.

### 4.5 HCMV and other cancers

Studies have shown that 77% of patients with prostate cancer have HCMV infection in their prostate tissues (Samsonov et al., 2012). In this study, the authors used PCR and *in situ* hybridization assays to detect HCMV infection. In another study, HCMV RNA was found in prostate cancer tissues using RNA sequencing methods (Chen and Wei, 2015). The presence of HCMV DNA in prostate cancer patients was confirmed with PCR-based detection (Martinez-Fierro et al., 2010). All evidences suggest a role for HCMV infection in prostate cancer progression. Similarly, HCMV has been associated with other types of cancer, such as medulloblastoma (Hortal et al., 2017), neuroblastoma (Wolmer-Solberg et al., 2013), sarcoma and mucoepidermoid cancer (Melnick et al., 2012; Jayaraj et al., 2015). Recently, HCMV DNA and proteins have been detected in tissue specimens collected from ovarian cancer patients (Xie et al., 2017; Carlson et al., 2018; Radestad et al., 2018; Ingerslev et al., 2019), suggesting a role for HCMV in this cancer. Several cancers that are believed to be caused by HCMV infection are described in **Table 3**.

## 5 Methods for treating HCMV infection

### 5.1 Chemical drugs

There are two main forms of treatments for HCMV infection-associated cancers: anti-viral therapy and immunotherapy. For antiviral therapy, small molecules such as ganciclovir, cidofovir, foscarnet, and fomivirsen still function as the standard of care in the clinical treatment of HCMV disease. Most of these drugs target DNA polymerase and block viral replication. Ganciclovir (GCV, Cytovene), which targets viral DNA replication, is the most widely used and is the first-line drug in the clinical treatment of HCMV infection (De Clercq, 2003). Up to now, the approved chemical drugs have failed to prevent HCMV infection in some conditions, such as lung, heart, pancreas, and allogeneic hematopoietic stem cell transplantation and immunodeficiency (Sia and Patel, 2000; Schreiber et al., 2009). Some adverse effects, including serious nephrotoxicity and neurotoxicity and drug resistance, still exist in clinical therapy. A safety and side effects study of ganciclovir in the treatment of HCMV infection was performed, and very few patients developed neurotoxic symptoms such as seizure or cerebral hypoxia(Kaul et al., 2011; Wong et al., 2016). This is a promising method for targeted therapy of HCMV-induced tumors (Solomon et al., 2014). Other chemical drug candidates have been reported in many studies and have the potential to become effective therapeutics for treatment of HCMV infection. AIC246 (letermovir), a novel chemical class of 3,4 dihydro-quinazolinyl-acetic acids, has been reported to be a well-tolerated anti-CMV agent that targets the viral terminase complex (an enzyme complex that plays a key role in the cleavage and packaging of CMV progeny DNA into capsides) and remains active against viruses resistant to DNA polymerase inhibitors. This agent could inhibit the formation and release of infectious virus particles (Kaul et al., 2011).

### 5.2 Gene therapy

siRNA (small interfering RNA), which has been emerged as a powerful tool in gene therapy, may provide a new way to fight viral infections. siRNAs recognize and bind to the target mRNA and in turn, reduce the expression of protein. It has been suggested that siRNA could be useful for the treatment of various diseases, such as cancer, neurodegeneration diseases, ophthalmic diseases and viral infection (Mansoori et al., 2014; Rupaimoole and Slack, 2017). Long-term treatment of HCMV infection with the most commonly used clinical drugs, such as GCV and its orally administered derivatives, is associated with some side effects (e.g., toxicity and drug resistance). Therefore, numerous studies have focused on the use of siRNAs in the treatment of HCMV infection. Target sites present throughout the entire infection cycle were selected as the targets of siRNA molecules. Two immediate-early proteins, IE1p72 and IE2p86, which are the first and most abundantly expressed proteins, are required during viral infection and replication. The IE1 gene (UL123) produces a 1.9-kb mRNA consisting of exons 1, 2, 3, and 4. The IE2 gene (UL122) produces an mRNA including exons 1, 2, 3, and 5. It has been reported that HCMV replication can be potently inhibited by transfection of siRNA into infected MRC5 cells (Shao et al., 2011; Hamilton et al., 2014). siRNAs targeting UL54 (DNA polymerase), UL97 (protein kinase), and UL122/123 (immediate early proteins) were examined at the cellular level for their ability to inhibit viral protein expression and viral replication. The results from this study indicated that the three selected siRNAs reduced viral progeny by 80.0%, 59.6% and 84.5%, respectively, at 7 days post-infection (Hamilton et al., 2014). It was also found that miR-US33-5p could inhibit viral DNA synthesis and viral replication. HCMV-miR-US33-5p could downregulate the expression of syntaxin3 (STX3), which is a soluble N-ethylmaleimide-sensitive attachment protein receptor involved in cellular membrane fusion and may function as a direct target site (Guo et al., 2015). Wang et al. investigated the relationship of HCMV-associated microRNA-613 with glioblastoma and reported that upregulation of microRNA-613 inhibited cell colony formation, invasion and migration (Wang et al., 2017). Gergen et al. reported that the anti-UL122/123 CRISPR/Cas9 system could reduce HCMV replication by targeting the viral genome and late-protein expression by 90%. In this study, the production of new viral particles was nearly eliminated and can serve as an effective method to clear CMV infection (Gergen et al., 2018).

### 5.3 HCMV-directed immunotherapy

Immunotherapy has emerged as a new approach for the treatment of HCMV-associated cancer. Antigens related to HCMV have been identified as potential targets in immunotherapy. For example, HCMV-associated proteins such as IE1, pp65, and late antigens could be detected in GBM tumor tissues with high specificity. These findings provide promising methods for HCMV-related cancer immunotherapy. Schuessler et al. developed a cellular immunotherapy method to treat GBM by targeting HCMV antigens in patients via autologous T cell therapy. The results demonstrated that the therapy was safe and promising for the treatment of GBM (Schuessler et al., 2014a). A study reported that HCMV phosphoprotein 65 (pp65) was expressed in more than 90% of GBM tissues and was not detected in normal brain tissue, suggesting that it may serve as a target for GBM therapy (Mitchell et al., 2008). By preconditioning the environment with the recall antigen tetanus/diphtheria (Td) toxoid before bilateral vaccination with DCs pulsed with pp65 RNA, patient survival and clinical outcomes were greatly improved (Mitchell et al., 2015).

It is indicated that CMV can function as an important immunotherapeutic target in cancer therapy for glioblastoma. Some studies have provided evidence that CMV antigen-pulsed autologous dendritic cells, autologous CMV-specific T cells, CMV-targeting CARs, and peptide vaccines are promising approaches for glioblastoma treatment (Nair et al., 2014a; Finocchiaro and Pellegatta, 2015). It has been reported that HCMV could be differentially detected in glioblastoma rather than normal brain tissues, making CMV antigens a potential target for glioblastoma multiforme (GBM) treatment. The therapeutic strategies based on the expression of CMV antigens may provide a feasible target for immunotherapy of HCMV-related cancers. HCMV develops several strategies to escape from the immune surveillance of the host. HCMV-directed immunotherapy has been used as a novel approach in HCMV-related cancers, and some clinical trials have been performed (Schuessler et al., 2014a). In one trial, pp65 was used as an immunological target for the treatment of HCMV infection (Bao et al., 2008). As shown in **Figure 2**, the generated immature DCs were transfected with CMV target-encoding mRNA and then matured using a standard maturation cocktail of GM-CSF and IL-4 with TNFα, IL-1β, IL-6, and PGE2. The results have indicated that patient-derived CMV pp65-specific T cells can recognize autologous target cells and provide an effective way to treat GBM (Nair et al., 2014b).

### 5.4 HCMV-specific immune response in humans

The protection against HCMV infection in pregnant women is a result of neutralizing antibodies, anti-gB antibodies, anti-PC antibodies, High avidity antibodies, CD4+ T cells, CD8+ T cells, CD4+, CD45RA+ T cells, and cytokines. Natural HCMV infection generates an effective broad immune response comprising the arms of both the innate immune system and adaptive and cellular immunity. During natural infection, the antibodies and T cells specific for PP65, IE1, IE2, and gB play an important role in inducing the immune response. However, sterile immunity against CMV infection has not yet been achieved. Studies have shown that gH/gL/pUL128-131 pentameric complex antibodies are crucial for protection against HCMV infection (Fouts et al., 2012; Chiuppesi et al., 2015; Ha et al., 2017).

The attenuated Towne vaccine generated by the extensive passages in tissue culture had inadequate efficacy in clinical trials (Plotkin et al., 1984; Plotkin et al., 1991). A two-base-pair mutation in the Towne UL130 gene is considered to be a major factor in the inadequate efficacy of the Towne vaccine in clinical trials (Dolan et al., 2004). Similarly, the AD169 vaccine strain has been shown to possess single nucleotide insertion in UL131 leading to a frameshift mutation (Wang and Shenk, 2005). The UL130 and UL131 genes encode functional UL130 and UL131 proteins that are essential to the development of an attenuated HCMV vaccine (V160), where a compound called Shield-1 is required for replication of the virus (Anderholm et al., 2016). The Shield-1 is a chemical compound that stabilizes a protein tagged with the destabilization domain (DD) and prevents proteasomal degradation. The DD is a mutated version of FkBP that causes the degradation of DD-tagged protein (Maynard-Smith et al., 2007; Madsen et al., 2020). The whole virus vaccine (V160) has been studied in clinical phase I trials and has been proven to be safe and immunogenic (Adler et al., 2019). Because the shield-1 ligand is not present in nature, V160 is unable to become replicative and thus has an excellent safety profile. Vaccines that are currently in trials are listed in **Table 4**. Recently, a live-attenuated replication-defective CMV vaccine using a chemical attenuated method was developed and proven to be effective in animal studies (Jaijyan et al., 2022a). This method of DNA virus attenuation using a chemical named centanamycin is a novel approach to combat DNA virus infection (Jaijyan et al., 2022a). CMV-based anti-aging gene therapy has been proven to be safe and effective suggesting CMV as a target of gene therapy for aging, cancer, and other diseases (Jaijyan et al., 2022b).

**Table 4:** A summary of the CMV vaccines in clinical studies.

CMV Vaccine	Design and construction	Current status
**AD169 vaccine**	The revertant Ad169 with restored pentameric complex and synthetic compound (Sheild-1)	Phase I
**Towne vaccine**	Virus attenuation due to a 2-bp (TT) insertion causing an aa frameshift mutation in UL130 Produced by125 passage in HELF	Phase I/II
**Town/Toledo chimera vaccine**	Produced by substituting genomic locuses of low passage Toledo strain with genomic region from attenuated Towne strain.	Phase I
**Alphavirus replicon particles (VRPs) vaccine**	An RNA based vaccine. The structural VEE virus protein genes were replaced with HCMV genes encoding for gB or pp65/IE1 fusion protein and VEE non-structural genes	Phase I
**Viral vectored HCMV vaccines**	Canarypox viral vectors used for HCMV antigens (gB, pp65) delivery.	Phase I
	Alphavirus vector used for HCMV antigens (gB, pp65) delivery.	Phase I
	Adenovirus type 6vector expressing IE-1, IE-2 and pp65	Phase I
**gB subunit vaccine**	gB	Phase II
**DNA-based vaccine**	ASP0113 vaccine contains two plasmid (VCL-6368, pp65 and VCL-6365, AD169 gB)	Phase I
	Trivalent DNA vaccine (VCL-CT02) encoding IE1,gB and pp65 antigens.	Phase I
**Pentameric complex vaccines**	gH/gL.pUL128L complex	Phase I
	MVA virus expressing 5 PC subunits	Mice
	PC vaccine produced in CHO cells	Mice
	A BAC cloned MVA vector expressing the 5PC subunits	Mice
	VRPs generated with VEE virus and expressing PC adjuvanted with MF59	Mice, Novartis vaccine
**PepVax vaccine**	HLA*201-pp65_495-503_	Phase I

### 5.5 Immune evasion in CMV

CMV uses multiple immune evasion strategies to escape from the host immune system. Natural killer (NK) cells are important components of the innate immune system. CMV inhibits NK cells via the CD94/NKG2 heterodimeric receptor by the host HLA-E pathway (Braud et al., 1998). Studies have shown that the viral UL140 protein binds to HLA-E and promotes its cell surface expression (Tomasec et al., 2000). Moreover, CMV expresses UL18, a viral homolog of cellular MHC class I molecules UL18, which are present on the surface of infected cells (Beck and Barrell, 1988). Furthermore, CMV UL18 binds to the inhibitory NK cell receptor LiLRB1 and inhibits MHC-class I binding to NK cells (Chapman et al., 1999). CMV-encoded UL16 inhibits the surface expression of the activating receptor ligands ULBP1, 2, 6 and MICB and thus inhibits NK cell activation (Eagle et al., 2009). CMV-encoded microRNA–UL112-1 also controls the activity of NK cells by supressiong the receptor-ligand MICB (Nachmani et al., 2010). There are many other CMV-encoded factors that inhibit NK cell signaling, thereby helping the virus escape from the host immune response (Lisnic et al., 2015; Hammer et al., 2018).

CMV displays several mechanisms to prevent CD8+ T cell recognition, including inhibition of MHC class I processing and presentation pathways. For example, US2 and US11 degraded MHC class I heavy chains (Jones et al., 1995; Wiertz et al., 1996), and US3 confined MHC class I peptide complex in the endoplasmic reticulum (ER) (Jones et al., 1996). CMV-encoded protein US6 blocks peptide translocation into the endoplasmic reticulum (Ahn et al., 1997; Kim et al., 2008). An elegant study by Cosman et al. in 2001 has suggested that CMV-infected cells may evade attack by host immune system by masking the recognition of ULBPs (UL16 binding proteins) and MICB (MHC class I antigen) via UL16 protein (Cosman et al., 2001). The ULBPs and MICB is serving as ligands for the NKG2D/DAP10 (activating receptors) and blocked by the soluble form of UL16 (Cosman et al., 2001).

CMV-encoded US2 inhibits MHC class II processing and presentation by degrading the HLA-DRα and HLA-DMα chains (Johnson and Hegde, 2002). CMV expresses UL111A during latency, a viral homolog of the immunomodulatory cytokine IL-10, and downregulates the expression of class I and class II MHC molecules (Spencer et al., 2002; Jenkins et al., 2008; Poole et al., 2020). It has been shown that CMV IL-10 aids in the persistence of latency by inhibiting allogeneic and autologous recognition by CD4+ T cells and helps the virus in evade the CD4+T cell response (Cheung et al., 2009). This indicates that CMV IL-10 plays an important role in the suppression of antigen presentation by latently infected cells. This evidence suggests that CMV has evolved to combat the host’s immune response and produce effective and latent infection.

### 5.6 CMV-induced miRNA

miRNAs are a class of noncoding RNAs of 20-24 nucleotides that post-transcriptionally regulate gene expression by forming the RNA-induced silencing complex (RISC) with an mRNA that possesses a complementary sequence. Many studies have shown that HCMV-encoded miRNAs target viral and cellular mRNAs for viral persistence and productive infection. CMV has been found to express miRNA and these CMV-encoded miRNAs were detected in the plasma (Mohammad et al., 2014), saliva (Hancock et al., 2012), and blood samples (Lisboa et al., 2015). Studies have revealed that the transcriptomic profile of HCMV-encoded miRNAs is not the same in different stages of the virus life cycle (Lau et al., 2016; Meshesha et al., 2016). The most studied miRNA of HCMV is mirUL112 which targets UL114 and plays an important role in regulating virus replication (Dunn et al., 2005). Moreover, the expression of miRNA in CMV has been associated with the establishment of viral latency in the host (Lau et al., 2016; Meshesha et al., 2016; Hancock et al., 2020). Studies have shown that HCMV-encoded miRUL36 can bind to the UL138 gene of HCMV and play a role in establishing viral latency (Hook et al., 2014).HCMV miR-UL12-1 controls the expression of the IE72 gene by targeting its 3’ UTR (Grey et al., 2007). HCMV miR-US5-1, miUS5-2, and miRUL112-3p regulate the expression of multiple genes in the endocytic recycling pathway and help virion assembly(Dangwal et al., 2012; Hook et al., 2014). Another study has revealed that HCMV-US25-2-3p binds with TIMP3 which is an important regulator in functions of NKG2D (natural killer group 2D ligand) which promotes cellular toxicity (Esteso et al., 2014). HCMV controls IL-6 secretion by targeting ACVR1B through HCMV-encoded miR-UL148D-1 (Lau et al., 2016) and targets RANTES/CCl5 (Kim et al., 2012).

Another interesting miRNA encoded by HCMV is CMV70-3P, which increases GBM CSC stemness. This miRNA can also control the expression of SOX2. The inhibition of CMV70-3P prevents glioma cells from proliferating and forming neurospheres (Ulasov et al., 2017). A list of miRNAs encoded by HCMV and cellular miRNAs is shown in **Table 5**. These miRNAs have been confirmed to control the various cellular processes that affect CMV replication and growth.

**Table 5:** A summary of CMV-induced miRNA.

Organism	miRNA	Target gene	description
HCMV	miR-UL112-1	UL17/18	MHC Class I homologue
		UL120/121	MIE region exons
		UL114	Viral DNA glycosylase
		IE72,	MIE IE72
	miR US5-1	US7	Unknown function
	miR US5-2	US7	Unknown function
Human	miR UL112-1	Transportin 1	Subunit of Karyoferin complex
		ZFP36L1	Zinc finger protein
		L7a	Ribosomal protein
		MICB	NK cell activating receptor
		IL32	cytokine
		SNAP23	Snaptosomal associated protein
	miR US25-1	TRIM28	Transcriptional corepressor
		NUCB2	Nucleobindin 2
		ATPV016	Component of vacuolar ATPase
		BCKDHA	Branched chain keto acid dehydrogenase E1
		SGSH	N-Sulfoglucosamine sulfohydrolase
		H3F3B	Histone H3 variant
		CCNE2	Cyclin E2
	miR US 5-2	CDC42	Cell division cycle 42
		SNAP23	Synaptosomal associated protein
	miR US 5-1	VAMP3	Cellubrevin
		RAB5C	Member RAS oncogene family
		RAB11A	cytokine release

### 5.7 Other methods

In addition to the methods mentioned above, there are some alternative therapies that may act as potential solutions for the treatment of HCMV infection. Several “on the shelf” drugs could be repurposed for CMV infection treatment. The PDGF receptor (PDGFR) serves as the most essential protein for cellular infection by the HCMV virion-containing trimeric complex. PDGFR is essential for the entry of the HCMV containing trimeric complex (gH/gL/gO) but not the pentameric complex (gH/gL/UL128-pUL130-pUL131A) (Wu et al., 2017). Moreover, PDGFR-α and the trimeric complex represent a predominant means of HCMV entry into fibroblast and pentameric complex facilitates the infection of endothelial as well as epithelial cells (Wu et al., 2018; Nishimura and Mori, 2019). Spiess et al. reported a new way to inhibit HCMV infection both *in vitro* and *in vivo* by designing a new HCMV-infected cell targeting system including a chemokine target molecule conjugated to a therapeutic fusion toxin (named F49A-FTP)(Spiess et al., 2017). The chemokine molecule binds to the G protein-coupled 7TM receptor US28, which expresses on the surface of HCMV-infected cells with high specificity and affinity. In this strategy, the chemokine functions as an anchor to guide toxin uptake by HCMV-infected cells, and both single doses and repeated doses of the chemokine conjugate F49A-FTP in a study, and resulted were more efficacious than GCV (Spiess et al., 2017). Apart from this, some chemical formulations can be applied in the treatment of HCMV infection. Russo et al. developed one kind of foscarnet-chitosan nanoparticles (Russo et al., 2014). Compared with free drugs, the encapsulated foscarnet showed a controlled drug release from nanoparticles and maintained antiviral activity with good performance against HCMV infection (AD-169 strain) in lung fibroblasts (HELFs). They used this method to improve delivery efficiency and reduce toxicity.

## 6 Clinical trials and implications

HCMV infection is a world-wide disease and can be latent in the host for their entire life, potentially causes consequences in pregnant women, and immune-suppressed patients. The illustration of a relationship between HCMV infection and carcinogenesis provided a new modality for cancer therapy. HCMV proteins such as IE1, (pp65) and (gB) have been suggested to function as potential immunotherapy targets (Vaz-Santiago et al., 2002; Meng et al., 2018). Strategies targeting these molecules could be used for the treatment of several types of cancer-related diseases, e.g. hematological malignancies, GBM, and gliosarcoma. Clinically, treatments of HCMV infection have been explored, and as recently as February 2017, there were 13 clinical trials regarding anti-HCMV therapy for GBM patients were conducted in the United States, and currently, there are numerous phase I and phase II studies that are still ongoing. Some of the clinical trials are registered on Clinicaltrials.gov and summarized in **Table 6**. Most of the results have been very encouraging and provide evidence that CMV infection can be a target for cancer therapy. Many clinical trials are currently active, and we do not have outcomes or results. One phase І study (NCT01109095, as listed in **Table 6**) reported the results of administering autologous CMV-T cells grafted with a HER2 chimeric antigen receptor to GBM patients. The cell product was generated from a peripheral blood draw of enrolled patients. In this study, the median survival of GBM patients administered with CMV pp65 specific T cells was 11.6 months from the infusion and 24.8 months from diagnosis (30 months from diagnosis for adults). This method was safe and a durable clinical effect for treatment was observed ~38% of patients (Ahmed et al., 2015). Another active clinical trial (NCT00400322, **Table 6**), which is being carried out in Karolinska University Hospital, is studying the efficacy and safety of Valganciclovir^®^ as an add-on therapy in patients with malignant glioblastoma and HCMV infection. This clinical trial was mainly based on the hypothesis that HCMV infection can promote tumor development and inhibit immune responses against the tumor. This ongoing study aimed to investigate whether treatment of CMV infection with the antiviral drug Valganciclovir^®^ affects the clinical outcome of GBM in patients with CMV infection (www.clinicaltrials.gov). Some additional studies involving in the use of HCMV as a target for cancer therapy have been completed, as shown on the Clinicaltrials.gov website. One study, entitled “Evaluation of recovery from drug-induced lymphopenia using cytomegalovirus-specific T-cell adoptive transfer”, aimed to evaluate whether vaccinating adult patients with newly diagnosed GBM who were seropositive for CMV using CMV-DCs during recovery from therapeutic TMZ-induced lymphopenia with ALT enhances the T cell response and safety. However, some trials, have been terminated, such as the trial entitled “Administration of CMV-Specific Cytotoxic T cells in Patients with Glioblastoma Multiforme” (NCT01205334) carried out by Baylor College of Medicine and the trial entitled “A Study Using Allogenic-Cytomegalovirus (CMV) Specific Cells for Glioblastoma Multiforme (GBM)” (NCT00990496), due to poor patient accrual rates. These results of clinical trials provide evidence that CMV can be used as a target for the treatment of cancer in spite of the mechanism by which HCMV is associated with cancers, such as GBM still needs to be investigated (Piper et al., 2021).

**Table 6:** Some clinical trials evaluating anti-HCMV infection therapy.

Studies	Sponsor	Status	Completion date	Registered NO.
Administration of CMV-Specific Cytotoxic T Cells in Patients With Glioblastoma Multiforme (COGLI)	Baylor College of Medicine	Terminated (poor accrual)	2018	NCT01205334
Peptide Targets for Glioblastoma Against Novel Cytomegalovirus Antigens (PERFORMANCE)	Gary Archer Ph.D.	Recruiting	2020^*^	NCT02864368
Efficacy and Safety of Valcyte^®^ as an Add-on Therapy in Patients With Malignant Glioblastoma and Cytomegalovirus (CMV) Infection	Karolinska University Hospital	Unknown	Not provided	NCT00400322
Autologous Cytomegalovirus (CMV)-Specific Cytotoxic T Cells for Glioblastoma (GBM) Patients	M.D. Anderson Cancer Center	Recruiting	2020^*^	NCT02661282
A Study Using Allogenic-Cytomegalovirus (CMV) Specific Cells for Glioblastoma Multiforme (GBM)	Milton S. Hershey Medical Center	Terminated (Accrual goals not met)	2010	NCT00990496
Peptide Vaccine for Glioblastoma Against Cytomegalovirus Antigens (PERFORMANCE)	University of Florida	Withdrawn (Investigator decided not to open study)	2014	NCT01854099
Analysis of CMV Infections in Patients With Brain Tumors or Brain Metastases During and After Radio(Chemo)Therapy (GLIO-CMV-01)	University of Erlangen-Nürnberg Medical School	Recruiting	2018*	NCT02600065
CMV-specific Cytotoxic T Lymphocytes Expressing CAR Targeting HER2 in Patients With GBM (HERT-GBM)	Baylor College of Medicine	Active, not recruiting	2014	NCT01109095

These results were posted on the website of United States Clinical trials. (https://clinicaltrials.gov/); *refers to the estimate complete time.

## 7 Conclusions

In recent years, the association between tumor progression and HCMV infection has been investigated. Cancer is a major health problem world-wide and causes millions of deaths every year. The aforementioned studies have shown that the HCMV genome contains oncogenes that have the potential to initiate various cancers. Studies have also confirmed the presence of HCMV-specific DNA and antibodies in tumors, indicating a direct role of HCMV-induced cellular transformation. Recently, several clinical trials have started to exploit HCMV as a therapeutic target for various cancers especially glioblastoma.

The key factors that HCMV induces cancer progression are still enigmatic, and concrete evidence is still lacking. There could be many unidentified genes in HCMV genome that contribute to cancer progression in host cells. Certainly, there is a need for investigators to address these gaps in knowledge as well as identify and characterize several unidentified genes to begin to fully understand the role of HCMV in tumor progression. Finally, a model of HCMV infection needs to be developed to study the gene-specific contribution to tumor progression, and the underlying mechanisms and biological pathways need to be elucidated.

## Author contributions

KG, CY, SH and XG conducted the review and wrote the draft. WZ and PR revised and designed the layout of the review. All authors contributed to the article and approved the submitted version.
